# Overuse injury induces persistent behavioral declines that correlate with higher IL-6 expression in the affected musculoskeletal tissues, circulation, and brain 

**DOI:** 10.3389/fphys.2025.1500795

**Published:** 2025-07-16

**Authors:** Mary F. Barbe, Alex G. Lambi, Michele Y. Harris, Parth R. Patel, Istvan P. Tamas, Elizabeth R. McGonagle, Megan Van Der Bas, Betsy A. Kalicharan, Lewis Bright-Rowe, Steven N. Popoff, David M. Klyne

**Affiliations:** ^1^ Aging + Cardiovascular Discovery Center, Lewis Katz School of Medicine of Temple University, Philadelphia, PA, United States; ^2^ Department of Surgery, Division of Plastic Surgery, University of New Mexico, Albuquerque, NM, United States; ^3^ The Hearth at Drexel, Liberty Lutheran, Lewis Katz School of Medicine of Temple University, and Community Life Leader II, Philadelphia, PA, United States; ^4^ NHMRC Centre of Clinical Research Excellence in Spinal Pain, Injury and Health, School of Health and Rehabilitation Sciences, The University of Queensland, Brisbane, QLD, Australia

**Keywords:** repetitive overuse injury, repetitive strain injury, musculoskeletal injury, work-related musculoskeletal disorders, pain, ependyma, cingulate cortex

## Abstract

**Background:**

Pain and sickness behaviors can be elicited by systemic inflammation. We sought to determine if mature rats displayed these behaviors following overuse injury and whether they correlated with inflammatory cytokines in musculoskeletal tissues, circulation, and the brain.

**Methods:**

Mature female Sprague–Dawley rats were used: 26 controls and 41 rats trained across 6 weeks to pull at high force levels. Following training, 28 rats performed a high-repetition low-force task for 6 more weeks (task); the remaining rested (trained + rest). Behavioral data were collected at baseline, following training, and at study end. Tissues and serum were then collected and examined for the presence of inflammatory cytokines.

**Results:**

Following training, task and trained + rest rats exhibited grip strength declines and forepaw sensitivity, compared to baseline and controls. Following task or rest, these behavioral changes persisted in addition to a reduction in social interactions (with juvenile female rats) in task rats, whereas trained + rest rats exhibited only low grip strength. Pro-inflammatory cytokines were elevated in serum and forelimb musculoskeletal and nerve tissues in task relative to control rats; IL-6 was elevated in serum and tissues in task relative to trained + rest rats. IL-6 immunostaining was observed in brain ependymal cells and cingulate cortex of task and trained + rest rats relative to control rats, and one circumferential blood brain region of task rats relative to the other groups. Higher cytokine levels in tissues often correlated with poorer behavioral responses.

**Conclusion:**

These data indicate that overuse injury induces inflammatory responses within the local/damaged tissues, circulation, and brain, which drives pain-related and sickness behaviors.

## 1 Introduction

Common upper-extremity work-related musculoskeletal disorders (WMSDs), also known as overuse injuries, repetitive strain injuries, and repetitive motion disorders, include peripheral mononeuropathies (e.g., carpal tunnel syndrome), tendon-related disorders (e.g., tendonitis), muscle-related disorders (e.g., myalgia), bone stress fractures, and reductions in bone density (Rempel and Diao, 2004; Sommerich et al., 2007; Szabo, 1998; van Tulder et al., 2007; Schorn et al., 1978; Vehmas et al., 2005). Chronic health conditions, including those with musculoskeletal symptoms in the hand and upper limb, have a major impact on absenteeism from work (sickness absences) (Bryan et al., 2021; Oke et al., 2016; Moreira-Silva et al., 2021). WMSDs are associated with early-onset and often progressive tissue inflammatory responses within the affected tissues with continued exposure to the inducing factor (i.e., work) (Fujiwara et al., 2017; Pinski et al., 2010; Barbe et al., 2013; Gold et al., 2016). These inflammatory responses include higher production of pro-inflammatory cytokines that enter into the circulation. For instance, individuals with upper-extremity WMSDs exhibit higher circulating levels of interleukin-1 alpha and/or beta (IL-1α/β), IL-1RII, IL-6, IL-18, tumor necrosis factor alpha (TNF-α), and C-reactive protein (CRP), compared to non-injured individuals (Carp et al., 2007; Gold et al., 2014; Gotoh et al., 2001; Matute et al., 2014; Rechardt et al., 2011), which correlate with symptom severity (Carp et al., 2007; Gold et al., 2014; Matute et al., 2014).

Pro-inflammatory cytokines are produced by numerous cells and have specific effects on the interactions and communications between immune-related cells, and play an integral role in the initiation, perpetuation, and up- or down-regulation of immune responses, depending on the cytokine (Zhang and An, 2007; Johnson et al., 1997). In addition to their roles in immune function, pro-inflammatory cytokines in peripheral tissues sensitize nociceptors either directly or by stimulating the release of agents that modulate nociception both peripherally and centrally (Zhang and An, 2007; Abbadie, 2005; Maier and Watkins, 2003; Watkins et al., 2003). Pro-inflammatory cytokines (namely, TNF-α, IL-6, and IL-1β) can also initiate and drive a range of physiological and behavioral responses known collectively as “sickness behaviors” (Dantzer et al., 1998; Henry et al., 2009; Kelley et al., 2003; Roth et al., 2021; Poon et al., 2013; Parnet et al., 2002). These “responses” include enhanced pain behaviors, reduced social interaction and exploration, and fatigue (Zhang and An, 2007; Bluthe et al., 2000a; Bluthe et al., 2000b; DeLeo and Yezierski, 2001; Queiroz et al., 2017; Dantzer et al., 2008), as well as aggression (Zalcman and Siegel, 2006; Suarez et al., 2002). Cytokines can enter the brain by crossing the blood–brain barrier (BBB) at specific sites (e.g., ependymal cells, endothelial cells, and circumventricular organs) or send signals that stimulate their expression in the brain via slow diffusion or stimulation of second messengers (Poon et al., 2013; Dantzer, 2001; Dantzer and Kelley, 2007; Capuron and Miller, 2011; Hoogl et al., 2015; Quan and Banks, 2007; Varatharaj and Galea, 2017). Once in the brain, these cytokines or signals can stimulate neurons and glial elements that also produce cytokines, express cytokine receptors, and amplify cytokine signaling, all of which can profoundly affect brain regions associated with sickness behaviors (Fasick et al., 2015; Dantzer, 2004).

Despite the known roles of cytokines in sickness responses and WMSD pathologies, their interrelationships are not typically considered together. This is important because psychosocial factors can contribute to pain persistence and are reported to predict symptom onset and outcome in individuals with WMSDs (Macfarlane et al., 2000; Theorell et al., 1991; Shaw et al., 2002; Harkness et al., 2004). Insights into whether these disorders stimulate psychosocial changes via inflammatory pathways might, in part, explain why psychosocial features often coexist with WMSDs (Macfarlane et al., 2000; Theorell et al., 1991; Shaw et al., 2002; Harkness et al., 2004; Cleland, 1987; Marinus and Van Hilten, 2006; Klyne et al., 2022). Furthermore, some cytokines are reciprocally linked with various psychological and behavioral factors, such as mood, anxiety, and sleep (Klyne et al., 2021; Klyne et al., 2024a; Klyne and Hall, 2024; Adcock et al., 2021; Felger, 2018; Irw and in, 2019; Krueger, 2008; Raison et al., 2006; Slavich and Irwin, 2014; Ulrich-Lai and Herman, 2009). Exposure to elevated pro-inflammatory cytokine levels may lead to depression (Raison et al., 2006; Slavich and Irwin, 2014; Capuron and Dantzer, 2003; Dantzer et al., 1999; Hayley et al., 2005). Conversely, stress-induced depression-like behaviors are associated with increased levels of systemic pro-inflammatory cytokines (Chang et al., 2024; Dowlati et al., 2010). These findings could also provide a potential explanation for the increased incidence of WMSDs and the decline in various psychosocial and behavioral aspects with aging (Krishnan et al., 2021; Collins and O'Sullivan, 2010). Underpinning this theory is the well-known increase in systemic inflammation that occurs with advanced aging, which is known as “inflammaging” (Fulop et al., 2023; Franceschi et al., 2020; Bektas et al., 2020; Batista et al., 2020; Franceschi et al., 2018a; Franceschi et al., 2018b; Monti et al., 2017). Whether the cycle of injury, inflammation, and sickness behaviors is amplified with aging is unclear.

Using a unique rat model of upper-extremity WMSDs, we have demonstrated that highly repetitive reaching and grasping tasks cause local nerve and musculoskeletal tissue injury, widespread increases in macrophage influx into musculoskeletal tissues and nerves, fibrosis in and around the median nerve, decreased nerve conduction velocity, and decreased grip strength in young adult and adult rats (i.e., aged 3–9 months) (Barbe et al., 2013). The macrophage response has a temporal correlation with increased cytokine levels in local (i.e., affected/damaged) musculoskeletal and nerve tissues and with increased circulating (serum) levels of several cytokines and chemokines (Barbe et al., 2013; Fisher et al., 2015). We have also reported evidence of sickness behaviors along with elevated serum and brain levels of pro-inflammatory cytokines in young adult rats (Xin et al., 2017). Moreover, we have observed grip strength declines that correlated with elevated inflammatory cytokines in circulation, and enhanced forepaw mechanical sensitivity concomitant with elevated TNF-α in median nerves and cervical spinal cord regions, in very mature rats (i.e., 14–18 months of age) performing a moderate-level repetitive task for 12 weeks (Elliott et al., 2010; Xin et al., 2011). However, we have not yet examined sickness behaviors in very mature rats and whether they relate to cytokines locally (i.e., in involved upper-extremity musculoskeletal tissues), systemically (i.e., circulation), and/or in the brain.

Although the role of cytokines at the site of peripheral tissue damage is well studied, understanding of systemic and brain cytokine responses and their interaction with pain and psychosocial behavioral (i.e., sickness) factors from the onset of upper extremity WMSDs is limited, especially in older adults. Nor is it known which (if present) sickness behaviors are exacerbated or persist, even with rest, in response to upper extremity WMSDs. Therefore, this study aimed to establish the following: 1) whether pain-related (grip strength and forepaw mechanical sensitivity) and sickness (reduced social interaction and increased aggression) behaviors are induced by highly repetitive reaching and grasping tasks over time in very mature rats (14 months of age at onset) using our established rat model of upper-extremity WMSDs; 2) whether these behavioral changes correlate with inflammatory cytokines released in peripheral tissues (musculoskeletal and nerve tissues), circulation (serum), and the brain. In addition, we explored the intensity (improve *vs.* worsen) and longevity (resolve *vs.* persist) of these responses over time in response to training and then rest, *versus* training and then task performance, by comparing rats that underwent 6 weeks of high intensity training to learn the task to induce the initial injury (“training”) followed 6 weeks of rest (“rest”), versus 6 weeks of low intensity yet more prolonged (per work day) reaching and grasping “task” after the initial training. We hypothesized that the training and task process would induce negative pain-related and psychosocial behaviors that correlate with elevated pro-inflammatory cytokine levels in local tissues, serum, and brain regions associated with cytokine trafficking into the brain and processing pain/sickness behaviors, and that these responses will be more pronounced in rats that went on to perform the task versus rest after training.

## 2 Materials and methods

### 2.1 Study design

**FIGURE 1:**
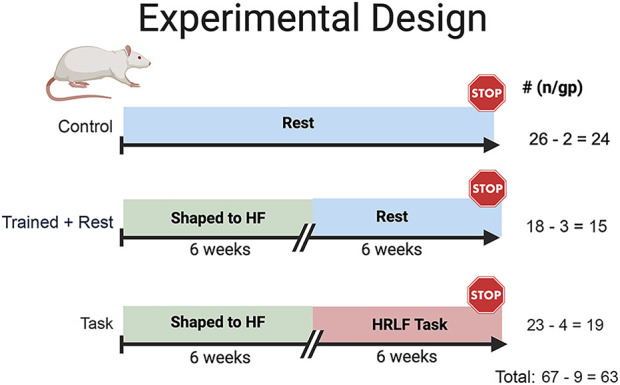
Experimental design. Studies began with 72 mature, female Sprague–Dawley rats (14 months of age at onset). All rats were placed on a food restriction (to ±5% of their naïve weight) for the duration of the experiment. Twenty-six rats were age-matched food-restricted controls (controls). Forty-six (18 trained + rest and 28 task rats) were trained under operant conditioning principles for 10–20 min/day, 5 days/week, for 6 weeks, to perform a reaching and lever-pulling task for a food reward until they were pulling the lever bar at 55% of their maximum voluntary pulling force. Twenty-eight of the trained rats went on to perform a high-repetition low-force task for 6 weeks (2 h/day, 3 days/week, at task requirements of four reaches/min and 15% of their max voluntary pulling force (task). The remaining trained rats rested for 6 weeks until tissue collection (trained + rest). Nine rats were excluded due to renal failure, tumors, or mortality, decreasing the final numbers per group, as indicated.

We used a three-arm randomized controlled design, as shown in Figure 1 and described further in Section 2.2.

### 2.2 Animals

Experiments were approved by the Institutional Animal Care and Use Committee in compliance with NIH guidelines for the humane care and use of laboratory animals. A total of 67 mature Sprague–Dawley rats (Figure 1) were procured at 9–10 months of age (Charles Rivers, Wilmington, MA, United States). Female rats were used for comparison with our past studies on similarly aged female rats using this same model (Elliott et al., 2010; Xin et al., 2011; Kietrys et al., 2012). In addition, since the force transducer sensitivity of our model setup is currently tailored to the pulling strength of female rats, the inclusion of male rats would have reduced data quality and made the interpretation of findings more difficult. We also focused on female rats because women have a higher incidence of work-related musculoskeletal disorders than men (Collins and O'Sullivan, 2010; Cote, 2012; Gerr et al., 2002). Rats were housed in an AAALAC-accredited central animal facility (while being handled daily, under 12-h light: 12-h dark cycle conditions, and with free access to water, 22–23°C room temp, cage changes 2/week, and filter topped cages). Rats were group-housed until experimental onset at 14 months of age. Thereafter, all rats were housed in individually transparent plastic cages due to the need for food restriction (necessary for motivation to perform the task as described below in Section 2.3). For environmental enrichment, all rats were handled daily for at least 30 min and were provided tunnels and chew toys.

Rats were numbered and randomly assigned to groups at the beginning of the study to ensure blinding. Forty-one rats were trained using operant conditioning principles to learn a high-force lever pulling task across 6 weeks, at no specified reach rate (Supplementary Figure S1). Of these rats, 23 performed a high-repetition low-force task for 6 weeks (task), while 18 trained rats rested (trained + rest) (Figure 1; Supplementary Figure S1). The remaining 26 untrained rats were maintained as age-matched controls and euthanized at the same time as either task or trained + rest cohorts. Rats were inspected weekly and post-mortem for the presence of illness or tumors. Data from nine rats were excluded from the study due to age-related health issues, e.g., renal failure (determined post-mortem by University Laboratory Animal Resource veterinarian staff), presence of tumors (identified visibly during the study and/or examined for post-mortem), or mortality due to unknown causes. These circumstances reduced the final group numbers to 24 controls, 15 trained + rest, and 19 task rats (Figure 1). Sentinel rats housed in the same room were examined monthly for the presence of viral or bacterial infections as part of the regular veterinary care (no infections were detected).

Not shown in Figure 1, ten juvenile (4–5 weeks of age) Sprague–Dawley female rats were included in the study to provide stimuli during social interaction behavioral testing, as described in Section 2.4. These rats were group-housed in cages separate from the mature adult rats. When these rats reached 6 weeks of age, they were excluded from this study and used for other purposes.

### 2.3 Injury induction and apparatuses

Once the mature rats reached 13.5 months of age and before the initiation of experiments, they were handled for 5–10 min each day for 2 weeks. Then, immediately prior to initiation of the experiments, all rats in the study were placed on a restricted diet to within ±5% of their naïve weights beginning at 14 months of age. This food restriction was necessary to motivate rats to work for a food reward. Control rats were similarly placed on a restricted diet as the task and trained + rest rats for the duration of the study. All rats were weighed at least once weekly throughout the duration of the experiment, and food was adjusted accordingly. In addition to food pellet rewards [banana (F0024) and chocolate (F0165) grain-based dustless precision pellets, Bio-Serv, Flemington, NJ, United States], all rats received Purina rat chow daily (5012, Purina, St Louis, MO, United States). Control rats received similar daily allotments of food reward pellets as task rats. Body weight at naïve (baseline) and study end was tracked and reported.

**FIGURE 2:**
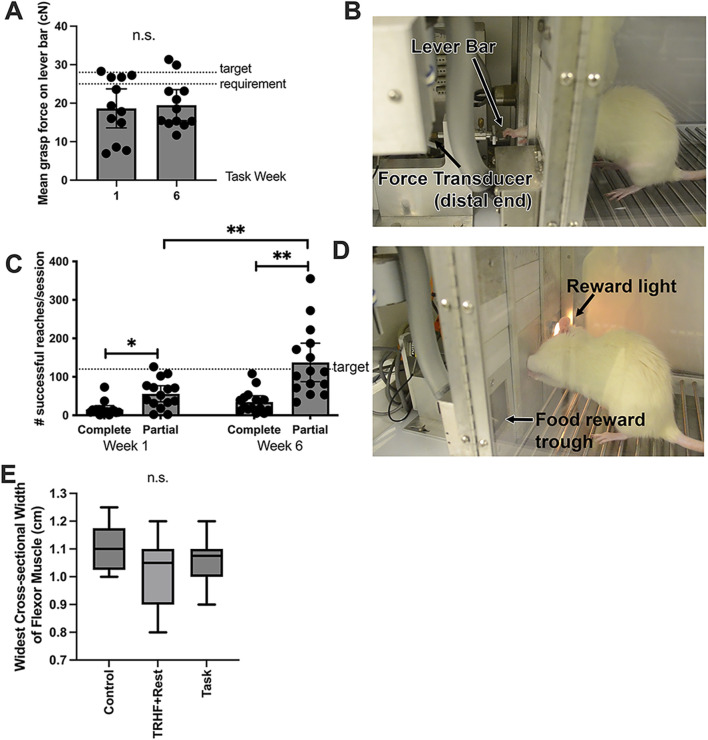
Voluntary grasp force and reaches per session at end of task weeks 1 and 6 and the widest cross-sectional width of the flexor digitorum muscle at the study end (task week 6). **(A)** Mean voluntary grasp force (in cN) on the lever bar by task rats (the only rats performing the task), which was required to be 26.4 ± 1.32 cN (15% ± 5% of their maximum voluntary pulling force, MPF, as indicated by the dotted lines in the graph) to receive a food reward. **(B)** Image of rat pulling on the lever bar, which is attached to a force transducer. **(C)** Number of reaches per session (successful and unsuccessful). The target reach rate was four reaches/min, as indicated by the dotted line in the graph, which is 120 reaches/session. **(D)** Image of a rat that just completed a successful reach, as indicated by the reward light. **(E)** Widest cross-sectional width of the proximal flexor digitorum muscle, shown as whisker plots that depict the mean, 95% confidence interval (grayed area), and standard deviation. This width was quantified from stained slides. n.s. = not significant. Symbols: * and ** represent p < 0.05 and p < 0.01, respectively, compared between groups as shown. Mean ±95% CI is shown.

Sixteen custom-designed behavioral apparatuses were used for the induction of upper-extremity WMSD, as shown in Figure 2 and previously described and depicted by Klyne et al. (2024b). Briefly, the task and trained + rest rats were first trained under operant conditioning principles to perform a progressively demanding reaching and lever-pulling task for 10–20 min/day (in the morning), 5 days/week, for 6 weeks, for a food pellet reward, as shown in Supplementary Figure S1. The handle of the lever bar was located 8.4 cm above the floor of the chamber, positioned 1.5 cm away from the chamber wall, and oriented horizontally. In their last week of training, the maximum voluntary pulling force exerted by the rats was assessed by removing the force lever program’s restrictions on the level of pull. The overall (all rats) mean maximum voluntary pulling force (MPF) was determined to be 176 ± 32 centiNewtons (cN). When the trained rats were able to independently pull on the lever bar at a high force level (55% ± 5% of the mean MPF, i.e., 96 ± 4.8 cN) at no particular reach rate, the training period ended (defined as task week 0).

Thereafter, 19 trained rats (randomly chosen) did not proceed to the task regimen, and instead rested for 6 weeks until euthanasia (trained + rest rats). The remaining 23 trained rats went on to perform a high-repetition low-force (HRLF) task regimen for 2 h/day in 30-min sessions separated by 1.5 h each (beginning at 9:30 a.m.), for 3 days/week (task rats only), at the target requirements of 4 reaches/min and 15% ± 5% of their mean MPF (26.4 ± 1.32 cN), for 6 weeks. The animals were not prevented from reaching at a higher or lower force than the target of 15% MPF. However, a food reward was not dispensed unless they initiated a reach within a 5-s window initiated every 15 s. They were allowed to reach with either or both limbs. The side(s) used to reach was recorded at each session. Thus, the animals were allowed to self-regulate their participation, making this a voluntary task. At the end of “task” or “rest” week 6, task and trained + rest rats were euthanized and tissues were collected (Figure 1). Control rats were euthanized at matched time points.

### 2.4 Behavioral testing

Prior to any behavioral measures, all animals were acclimated to the assay apparatuses and experimenters for at least 2 weeks, with at least 30 min of acclimation to each assay apparatus, prior to any data collection. Behaviors were measured at baseline, at the end of task weeks 0 or 1 (the latter for reach performance data), and at week 6 in n = 15–24/group. These behavioral assays were carried out between 1 and 5 p.m. on non-task days (to avoid rat fatigue). Limbs used for reaching were tracked at each session. Data are reported from the primary limbs used to reach (either right, left, or the average of both) by each rat for reflexive grip strength and forepaw mechanical sensitivity. Behavioral assays were performed by an examiner blinded to group assignment.

Force lever data were recorded continuously during each task session (i.e., for task rats) for later calculation of voluntary grasp force and reach rate via an automated script (MTLAB and Mathworks, Natick, MA, United States), as described previously (Barbe et al., 2013). A reach was defined as a force defection that exceeded 2.5% of the baseline (or zero) force until the next sample in which the force fell below 2.5% of baseline. In this way, unsuccessful reaches that did not meet the food reward criteria were recorded and analyzed along with successful reaches. The number of successful versus unsuccessful reaches per session is reported. Data from the end of the first task week (i.e., week 1 of task; the first week rats performed the task regimen for 2 h/day, 3 days/week) were used as the baseline for reach performance and compared to week 6 data.

Reflexive grip strength of the forelimbs was tested bilaterally using a grip strength meter for rodents (0167-8002, Columbus Instruments, Columbus, Ohio, United States), as previously described (Klyne et al., 2024b). Briefly, animals were first acclimated to the grip strength testing instrument for several days (at least 5 min per day for 3 days) prior to the day of the actual testing and data acquisition. The software of the testing device calculated the data, reducing potential human error. The moment at which each animal released its grip from the handle of the grip strength meter was self-determined and subject to factors such as muscle myalgia (Schafers et al., 2003). The test was repeated five times per forelimb, in a randomized fashion, and the maximum reflexive grip force (in cN) per trial is reported.

To test forepaw/forelimb withdrawal behaviors, the animals were habituated to a clear plastic chamber above a metal mesh (0.5 × 0.5 cm^2^) prior to the onset of the experiments. On the day of the experiment, rats were placed into a clear acrylic chamber with a metal grid floor and allowed to acclimate to this environment for at least 10 min. A series of calibrated nylon monofilaments (58011, Semmes–Weinstein monofilaments, Stoelting, Woodale, IL, United States) were applied from below to the center of the glabrous surface of the forepaws, using previously described methods (Clark et al., 2004). Filaments were applied to one forepaw at a time to the mid-palmar surface of the paw through the mesh floor until the filament bent slightly and was kept in this position for approximately 5 s, beginning each time with the lowest-sized filament and then sequentially applying the probe once to the right forepaw of all rats before applying the probe once to the left forepaw of all rats. Each filament test was repeated five times per session for each forepaw, and the animal was allowed to rest for approximately 3 min between each of the five trials per filament and per forepaw. The smallest monofilament that elicited a forelimb withdrawal in each of the 5 applications was considered the threshold stimulus. The force at which this monofilament bent is reported in cN.

Social interaction time was assessed by observing the duration of social exploration between a novel juvenile rat placed into a clean testing cage with the mature rat (which had been acclimated to this arena at least 5 min before introducing the juvenile rat) using described methods (Xin et al., 2017; Barbe et al., 2024). Briefly, the duration was assayed during the first 5 min following the introduction of the juvenile rat. Positive interactions with the juvenile rat included: 1) sniffing of any kind (face-to-face or genitoanal), 2) crawling over or under the younger rat, 3) grooming, and/or 4) licking the juvenile rat. The duration of these interactions was tracked using a stopwatch and is presented as the total number of seconds that the mature (experimental) rat spent interacting positively (as defined above) with the juvenile rat across the testing time-frame. Aggression (boxing, lateral displays, biting, and/or kicking) displayed by the mature rat toward the juvenile rat was also recorded. Such actions ended the social interaction session, and the juvenile rat was removed from the cage immediately. The proportion of rats showing one or more aggressive behaviors is presented.

### 2.5 Tissue collection

Task and trained + rest rats were euthanized at 6 weeks after the onset of task performance or rest, respectively. Control rats were euthanized at matched time-points. For this, at 18 h after completion of the final task session (to avoid acute increases in serum cytokine levels associated with task performance), animals were deeply anesthetized with 5% isoflurane in oxygen. Depth of anesthesia was assessed and monitored by the pattern and rate of respiration, the absence of muscle tone, and the absence of toe and tail pinch and eye blink reflexes. When the animals no longer showed any reflexive responses, showed an absence of muscle tone, and breathing had halted, they were subjected to a thoracotomy and cardiac puncture with exsanguination.

For biochemical assays, blood was collected by cardiac puncture using a 18-gauge needle and centrifuged, and the supernatant was collected as serum and frozen for later ELISAs from n = 12–22/group using described methods (Xin et al., 2017; Xin et al., 2011). Flexor digitorum muscles, flexor digitorum tendons, forelimb bones (radius and ulna), and median nerves were dissected bilaterally, collected, and flash-frozen to be used for later ELISAs from n = 6–12/group. The tissues were collected separately, rinsed in sterile saline, dissected into smaller samples of approximately 50–100 mg each, and flash-frozen in liquid nitrogen before storage at −80°C. Tails were also collected, bone and tendons removed, and analyzed, to examine non-loaded tissues. Unfixed brains were collected from 6 to 7 animals per group. These brains were dissected quickly into cingulate, hindbrain, and forebrain regions and flash-frozen in liquid nitrogen before storage at −80°C until used later for ELISA.

For histology and immunohistochemical analyses, rats underwent intracardial perfusion with first saline and then buffered 4% paraformaldehyde. Flexor digitorum muscles were then collected from n = 8–13/group, and brains were collected from n = 5–12/group. Flexor digitorum muscles were collected bilaterally. The tissues were fixed by immersion overnight in buffered 4% paraformaldehyde and stored first in 10% and then 30% sucrose in phosphate buffer (pH 7.4) for 48 h each before being embedded in Optimum Cutting Temperature compound (23730571, FisherScientific, Houston, TX, United States) on dry ice. Embedded tissues were stored at −80°C until cryosectioned.

### 2.6 ELISAs

Serum, forelimb musculoskeletal tissues, and median nerves were assayed to detect levels of IL-1α, IL-1β, IL-6, IL-10, and TNF-α using previously described methods (Xin et al., 2017; Xin et al., 2011) and commercially available ELISA kits (IL-1α - BMS627, IL-1β - BMS630, IL-6 - ERA31RB, IL-10 - ERA23RB, and TNF-α - KRC3011; BioSource, Invitrogen Life Sciences, CA, United States). We chose to assay three key pro-inflammatory cytokines, IL-1α, IL-1β, and TNF-α; a potent anti-inflammatory cytokine, IL-10; and IL-6, which is considered both pro- and anti-inflammatory. Serum data are presented as pg/mL serum. Brain tissues (cingulate cortex, hindbrain, and forebrain) were also assayed for IL-6 in select animals (animals not perfused with fixative). Tissue ELISA data were normalized to the total protein concentration as measured using BCA protein assays (23227, Pierce, Thermo Fisher Scientific, Rockford, IL, United States) and then expressed as pg/microgram of the total protein. All serum and tissue samples were analyzed in duplicate in a blinded fashion and batched where possible to reduce potential inter-assay variability. Data from primary reach limbs were used for data analyses. For animals that reached with both limbs, data from the right and left limbs were averaged before data analysis.

### 2.7 Histological and immunohistological assays

The proximal portion of flexor digitorum muscles was cryosectioned into cross-sections that were 14 μm thick and placed onto charged slides, with two sections per slide (22037200, Fisher Scientific, Pittsburgh, PA, United States). Sections on slides were dried overnight at room temperature before storage in foil-wrapped slide boxes at −80°C until use. These sections were stained with hematoxylin and eosin. The widest parts of each proximal portion of flexor digitorum muscle were quantified in order to assay for possible atrophy or gain in the muscle. At least three cross-sections were assayed per muscle, and the greatest width reported in centimeters (cm).

Forebrain and mid-brain regions were similarly cryosectioned into 14-μm-thick cross-sections. Sections on slides were dried overnight at room temperature before storage in foil-wrapped slide boxes at −80°C until use. Sections on slides were blocked with 4% milk in 10% goat serum diluted with 0.1% Triton X-100 in PBS for 1 h at room temperature using a specific anti-rat IL-6 antibody (ab9324, Abcam, Waltham, MA, United States 1:500 dilution in PBS). After washing, the primary antibodies were visualized using appropriate secondary antibodies (all from Jackson ImmunoResearch Laboratories, West Grove, PA, United States) conjugated to either horseradish peroxidase (HRP) or Cy2 (green fluorescence) and diluted 1:250 in PBS for 2 h at room temperature before washing. The HRP tag on secondary antibodies was visualized using diaminobenzidene (brown) (SigmaFast D4293-50SET, St. Louis, MO, United States).

To examine which cell types were expressing IL-6, double-labeling was performed by combining the IL-6 antibody with an antibody to a specific marker: NeuN (a specific neuron marker; MMB337, Millipore, Billerica, MA, United States, 1:200 dilution in PBS), glial fibrillary acidic protein (GFAP; a specific astrocyte marker; ab7260, Abcam, 1:500 dilution in PBS), or Iba-1 ((ionized calcium-binding adapter molecule 1, a specific microglial cell marker; ab15690, Abcam, 1:50 dilution in PBS). These latter primary antibodies were visualized using appropriate secondary antibodies (from Jackson ImmunoResearch Laboratories) conjugated to Cy3 (red fluorescence) and diluted 1:250 in PBS for 2 h at room temperature before washing.

Brain regions examined for IL-6 immunoreactivity included the ependyma surrounding the lateral and third ventricles, rostral cingulate cortex, vascular organ of the lamina terminalis (OVLT), subfornical organ (SFO), and median eminence. Images were captured with either a Nikon E600 (bright field) or Nikon E800 (epifluorescent scope) (Nikon Instruments, Melville, NY, United States). The number of labeled cells per region of interest with cytokine immunoreactivity was quantified using previously described methods (Elliott et al., 2010). Briefly, three measurements were made in the region of interest, per section, in at least two sections/rat, with each quantified section separated by at least 140 μm from other quantified sections. Measurements were made using an irregular ROI (e.g., for the ependyma) or a set square area of 13.3 cubic microns, a 40X objective, and an image analysis system (Nikon E800 with a Jenoptik Gryphax digital camera, interfaced to a Dell Computer (Windows 11) with the Life Science program by BIOQUANT, Nashville, TN, United States). Data were exported to Microsoft Office Excel and averaged per region per rat brain. Results are expressed as the mean number of IL-6+ cells/mm^2^. Numbers of IL-6+ cells that were also neurons (NeuN), astrocytes (GFAP+), or microglia (Iba-1+) were also recorded using a 40X objective for select brain regions. These assays were performed by an examiner blinded to group assignment.

### 2.8 Statistics

The sample size for this study was derived from our previous studies involving mature rats (Elliott et al., 2010; Xin et al., 2011; Kietrys et al., 2012). From these data, a power analysis set at conservative thresholds of 80% power and a 0.05 alpha level indicated that at least five rats/group were needed for histological and biochemical analyses. Therefore, in this study, similar or more animals per group were analyzed: *n* = 5–22/group for biochemical analyses, *n* = 5–12/group for brain histological analyses, and *n* = 11–25/group for behavioral analyses, with the exact number indicated in the scatterplot bar graphs. A *post hoc* power analysis confirmed that 80% power was maintained for each analysis per group. For example, it confirmed that at least n = 11/group was needed for the grip strength analyses, at least n = 5/group for duration of social interaction, at least n = 3/group for the serum IL-1 and IL-6 results, and at least n = 5/group for the IL-6 immunostaining in the brain ependymal cells.

GraphPad Prism version 10 was used for statistics and graphing. Data are represented graphically as mean ±95% confidence intervals (CI). P-values <0.05 were considered statistically significant. P-values reported for *post hoc* findings are adjusted for the number of multiple comparisons performed. Repeated-measures mixed-effects models (REML, restricted maximum likelihood) with Geisser–Greenhouse corrections were used to compare grip strength, mechanical sensitivity, and duration of social interaction using the factors *group* and *time* in the study (baseline, week 0, and week 6). Similar repeated-measures mixed-effects models were used to examine differences in serum and tissue cytokine levels using the factors *group* and *cytokine*. These were followed by Tukey’s multiple comparison *post hoc* tests. A repeated-measures mixed-effects model was also used to compare successful versus unsuccessful pulls performed by task rats in weeks 1 versus 6. Welch’s t-test was used to compare voluntary grasp force by task rats in weeks 1 versus 6. One-way ANOVA was used to compare muscle widths between groups. A chi-square contingency test was used to examine group differences in the incidence of aggression. Pearson’s or Spearman’s rank correlation tests were used, as appropriate, to assess the relationships between measures. Values between ±0.3 and ±0.59 were considered to indicate a moderately positive or negative relationship, those between ±0.6 and ±0.79 were considered to indicate strong relationships, and those between ±0.8 and 1.0 were considered to indicate very strong correlations (Swinscow et al., 1997; LaMorte, 2019). However, these values are arbitrary limits, and correlation results should be considered in context.

Scatter dot graphs of behavioral data are shown in Supplementary Figure S2. *Post hoc* results are shown in figures, and correlations are shown in Tables 1–4.

**TABLE 1:** Correlations between serum cytokines.

Serum cytokine	Serum IL-1α	Serum IL-1β	Serum IL-6	Serum TNF-α
IL-1α	—	r = 0.06, p = 0.35	r = 0.08, p = 0.28	**r = 0.54, p < 0.0001**
IL-1β	r = 0.06, p = 0.35	—	r = 0.15, p = 0.18	**r = 0.70, p < 0.0001**
IL-6	r = 0.63, p = 0.003	r = −0.07, p = 0.39	—	r = −0.22, p = 0.15
TNF-α	**r = 0.54, p < 0.0001**	**r = 0.70, p < 0.0001**	r = 0.08, p = 028	—

Spearman’s r values are shown. Significant correlations are bolded.

**TABLE 2:** Correlations between serum cytokines with same cytokines in forearm tissues.

Forelimb tissue	Serum IL-1α	Serum IL-1β	Serum IL-6	Serum TNF-α
Forelimb flexor muscles	**r = 0.43, p = 0.02**	r = 0.16, p = 0.27	**r = 0.53, p = 0.008**	r = −0.14, p = 0.27
Forelimb flexor tendons	r = −0.30, p = 0.10	r = 0.29, p = 0.15	r = 0.35, p = 0.07	r = −0.21, p = 0.19
Forelimb bones (radius and ulna)	**r = 0.63, p = 0.003**	r = −0.07, p = 0.39	**r = 0.39, p = 0.03**	r = −0.22, p = 0.15
Median nerves	**r = 0.49, p = 0.008**	r = −0.25, p = 0.16	**r = 0.53, p = 0.01**	**r = 0.45, p = 0.01**

Spearman’s r values are shown. Significant correlations are bolded.

**TABLE 3:** Correlations between serum/tissue cytokines and behaviors.

Serum and forelimb tissues	IL-1α	IL-1β	IL-6	TNF-α
Reflexive grip strength^a^				
Serum	**r = -0.35, p = 0.02**	r = 0.12, p = 0.45	**r = -0.40, p = 0.008**	r = −0.05, p = 0.68
Flexor muscles	**r = -0.55, p = 0.007**	r = −0.08, p = 0.75	**r = -0.50, p = 0.02**	r = 0.04, p = 0.86
Flexor tendons	**r = 0.58, p = 0.01**	**r = 0.51, p = 0.02**	r = −0.22, p = 0.34	r = 0.003, p = 0.88
Forelimb bones	**r = -0.42, p = 0.03**	r = −0.38, p = 0.06	**r = -0.45, p = 0.02**	r = −0.02, p = 0.93
Median nerves	r = −0.29, p = 0.20	**r = -0.66, p = 0.004**	**r = -0.63, p = 0.005**	**r = -0.69, p = 0.001**
Forepaw withdrawal threshold (mechanical sensitivity)^b^				
Serum	r = 0.10, p = 0.26	r = 0.16, p = 0.18	**r = -0.50, p = 0.003**	r = 0.20, p = 0.10
Flexor muscles	r = −0.14, p = 0.30	r = 0.17, p = 0.26	r = −0.19, p = 0.23	r = −0.09, p = 0.49
Flexor tendons	r = −0.09, p = 0.34	r = 0.19, p = 0.25	r = −0.03, p = 0.45	r = 0.46, p = 0.09
Bones	r = −0.26, p = 0.15	r = 0.06, p = 0.42	r = −0.32, p = 0.10	r = −0.06, p = 0.40
Median nerves	**r = -0.51, p = 0.007**	**r = -0.51, p = 0.009**	**r = -0.56, p = 0.003**	**r = -0.49, p = 0.01**
Duration of social interaction in a positive manner^a^				
Serum	r = −0.06, p = 0.93	r = 0.33, p = 0.06	**r = -0.50, p = 0.001**	r = 0.004, p = 0.79
Flexor muscles	r = −0.25, p = 0.25	r = 0.10, p = 0.64	**r = -0.53, p = 0.01**	r = 0.06, p = 0.79
Flexor tendons	r = −0.35, p = 0.10	r = −0.10, p = 0.65	r = −0.05, p = 0.81	r = −0.37, p = 0.08
Bones	**r = -0.42, p = 0.04**	r = 0.09, p = 0.64	r = −0.12, p = 0.38	r = −0.25, p = 0.22
Median nerves	r = −0.40, p = 0.08	r = −0.45, p = 0.06	r = −0.15, p = 0.27	r = −0.27, p = 0.12
Incidence of aggression during social interaction testing^b^				
Serum	r = 0.07, p = 0.69	r = −0.20, p = 0.34	**r = 0.45, p = 0.01**	r = 0.22, p = 0.24
Flexor muscles	r = 0.30, p = 0.19	r = 0.01, p = 0.94	**r = 0.57, p = 0.008**	r = −0.11, p = 0.64
Flexor tendons	r = 0.13, p = 0.87	r = −0.06, p = 0.79	r = −0.16, p = 0.50	r = 0.21, p = 0.38
Bones	**r = 0.42, p = 0.04**	r = 0.04, p = 0.86	r = 0.04, p = 0.85	r = −0.09, p = 0.65
Median nerves	**r = 0.50, p = 0.04**	r = 0.16, p = 0.57	r = 0.26, p = 0.33	r = 0.33, p = 0.19

^a^ Pearson’s r and p values are shown. Significant correlations are bolded.

^b^ Spearman’s r and p values are shown. Significant correlations are bolded.

**TABLE 4:** Correlations between brain IL-6 and behaviors.

Brain region	Immunostained cells^a^	Grip strength^a^	Forepaw withdrawal threshold^b^	Duration of social interaction^a^	Aggression^b^
Cingulate cortex	IL6+NeuN+ (neurons)	**r = -0.38, p = 0.03**	**r = -0.57, p = 0.002**	**r = -0.66, p = 0.007**	r = 0.49, p = 0.07
Cingulate cortex	IL6+Iba-1+ (microglia)	r = 0.13, p = 0.30	r = −0.13, p = 0.30	r = 0.20, p = 0.29	r = 0.17, p = 0.33
Ependymal cells	IL6+ (ependymal cells)	**r = -0.59, p = 0.001**	**r = -0.60, p = 0.001**	**r = -0.47, p = 0.04**	r = 0.23, p = 0.25

^a^ Pearson’s r and p values are shown. Significant correlations are bolded.

^b^ Spearman’s r and p values are shown. Significant correlations are bolded.

## 3 Results

### 3.1 Successful reaches declined with continued task performance

As the rats self-regulated their involvement in the “voluntary” reaching and pulling task (i.e., rats choose to participate or not at their own intensity), they tended to reach and pull differently than the assigned target requirements. We assessed this variation in task rats by examining the mean voluntary grasp force applied to the lever bar and the number of unsuccessful reaches (i.e., did not meet the target force requirement or not within the correct time frame) vs. successful (met the target of 15% MPF within the time requirement) for weeks 1 and 6 (Figures 2A–D). With respect to grasp force, only some task rats pulled the lever bar at or near the target of 15% MPF of 26.4 ± 1.32 cN in either task weeks 1 or 6, and there was no difference between weeks (Figure 2A). However, the number of successful and unsuccessful reaches differed between weeks (p < 0.0001, F_1,56_ = 28.70). *Post hoc* analysis revealed more unsuccessful reaches than successful reaches at both time points (Figure 2C). The number of unsuccessful reaches was greater than the target of 120 reaches/session, particularly in task week 6 compared to task week 1. This suggests that rats did not rely completely on auditory or light prompts to initiate reaching tasks, which may indicate that they did not effectively learn that a food reward could only be obtained four times per minute and, therefore, pulled the lever bar more frequently (i.e., above the target reach rate) in an attempt to obtain food rewards.

### 3.2 Flexor digitorum widths did not alter with task or training

Flexor digitorum muscle widths were examined for indices of possible muscle atrophy or hypertrophy in response to training or task performance. No differences were observed (Figure 2E).

### 3.3 Training-induced behavioral declines were exacerbated with continued task performance; only grip strength declines persisted in trained + rest rats

**FIGURE 3:**
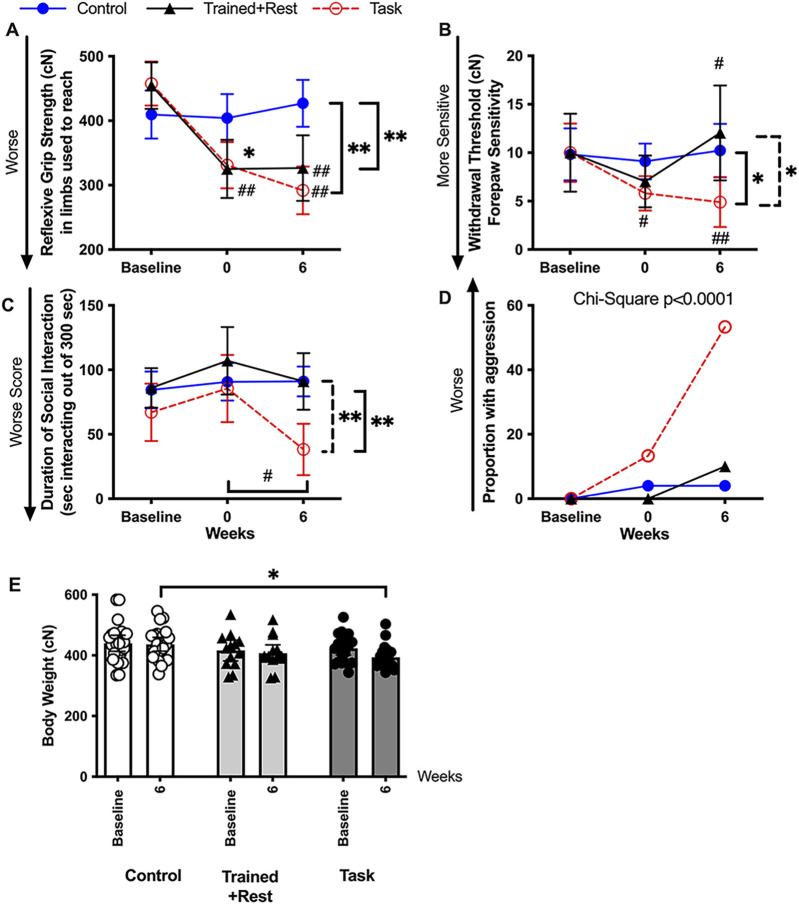
Pain-related and psychosocial (sickness) behaviors at baseline, 0, and 6 weeks. **(A)** Forearm reflexive grip strength. **(B)** Forepaw sensitivity assayed as smallest-sized monofilament that elicited a forelimb withdrawal response over five trials. **(C)** Duration in which the experimental mature rats socially interacted in a positive manner with a novel juvenile rat. **(D)** Proportion of experimental rats showing aggressive behaviors. **(E)** Body weight (in cN) for each group at naïve and task week 6 (or equivalent time point). n.s. = not significant. Symbols: # and ##: p < 0.05 and p < 0.01, respectively, compared to baseline. * and **: p < 0.05 and p < 0.01, respectively, compared between groups as shown. Mean ±95% CI is shown. Tr + rest = trained + rest.

Reflexive grip strength is both a motor and a muscle myalgia assay (Fujiwara et al., 2017; Schafers et al., 2003) and is a proposed biomarker of aging (Bohannon, 2019). Mixed-model analysis revealed differences between *groups* (p = 0.002, F_2,56_ = 7.04), *time* in study (p < 0.0001, F_2,95_ = 25.45), and a significant *group × time* interaction (p < 0.0001, F_4,104_ = 9.10). *Post hoc* analysis revealed declines in forelimb withdrawal thresholds in both Trained+Rest and Task rats in weeks 0 and 6, compared to baseline and Control rats (Figure 3A; scatter plots are shown in Supplementary Figure S2A).

Forelimb withdrawal thresholds are a measure of the mechanical sensitivity, with lower thresholds indicative of enhanced forepaw mechanical sensitivity (Adcock et al., 2021; Tal and Bennett, 1994). Mixed-model analysis revealed differences in *time* in study (p = 0.03, F_2,98_ = 3.51) and a significant *group × time* interaction (p = 0.04, F_4,98_ = 2.57). *Post hoc* analysis revealed declines in forelimb withdrawal thresholds in both trained + rest and task rats in week 0 compared to baseline and control rats (Figure 3B; Supplementary Figure S2B). Forelimb withdrawal thresholds had resolved to baseline levels in trained + rest rats but persisted in task rats following the 6-week rest and task period. This finding was reflected by lower forelimb withdrawal thresholds in task rats in week 6 compared to both other groups (Figure 3B; scatter plots are shown in Supplementary Figure S2B).

The social interaction test measures the sociability and depressive-like or anxiety-like behaviors, with more time spent positively interacting with another rat indicative of sociability and preference for social novelty and less time spent indicative of less sociability, depression, or anxiety (Bluthe et al., 2000a; Klyne et al., 2024b; Kim et al., 2019). Mixed-model analysis revealed differences in the duration of positive social interactions by *group* (p = 0.002, F_2,48_ = 7.37), *time* in study (p = 0.005, F_1,847_ = 5.98), and their interaction (p = 0.03, F_4,78_ = 2.95). *Post hoc* analysis revealed significant declines in the duration of a positive social interaction in 6-week task rats compared to their baseline, trained + rest rats and control rats (Figure 3C; scatter plots are shown in Supplementary Figure S2C). Trained + rest and control rats did not differ over time. A substantially greater proportion of task rats exhibited aggressive behaviors in task week 6 relative to both other groups (p < 0.0001, chi-square df_44,8_; Figure 3D; proportion as bar graph is shown in Supplementary Figure S2D).

In addition, body weight was tracked across time for each rat. As shown in Figure 3E, task rats weighed slightly, yet significantly, less than control rats in task week 6 (also shown in Supplementary Figure S2E).

### 3.4 Pro-inflammatory cytokine production was elevated in serum and tissues involved in task performance

We previously reported that circulating levels of IL-6 and IL-1α are elevated in mature rats following 12 weeks of performing a similar high-repetition low-force task and that their elevations correlated with grip strength declines (Xin et al., 2011). We extended those findings here to determine if pro-inflammatory cytokine levels are also elevated in the circulation (serum) and forearm tissues involved in task performance (i.e., flexor digitorum muscles and tendons, forearm bones, and median nerves) following 6 weeks of task performance relative to training only (trained + rest) or neither (control).

**FIGURE 4:**
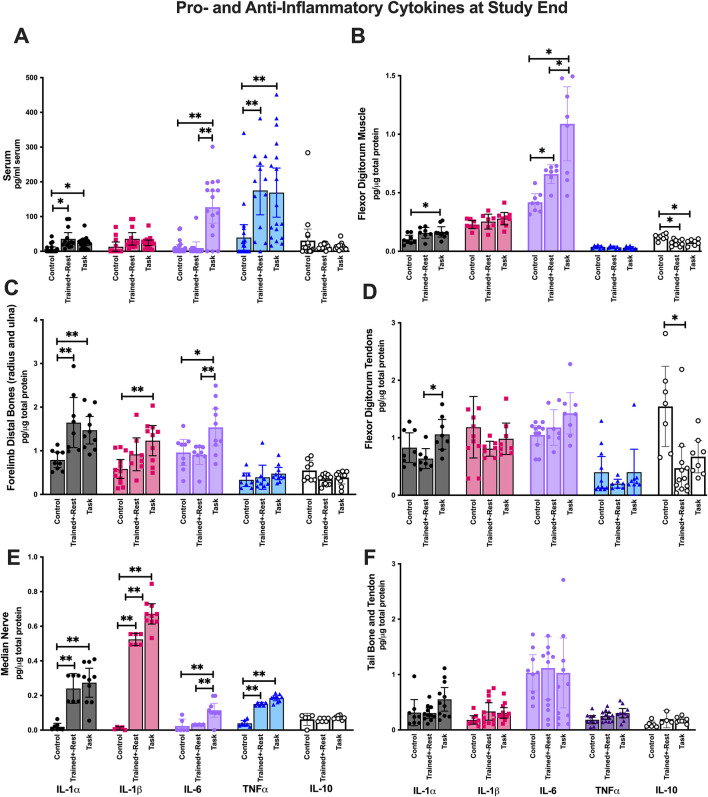
Pro- and anti-inflammatory cytokines in serum, forearm tissues involved in the reaching and pulling task, and tail (i.e., uninvolved) tissues collected after euthanasia. Each was tested using ELISA for the inflammatory cytokines indicated. **(A)** Serum. **(B)** Forearm flexor digitorum muscles. **(C)** Forearm bones (radius and ulna combined). **(D)** Forearm flexor digitorum tendons. **(E)** Median nerves. **(F)** Tail bones and tendons combined. Symbols: * and **: p < 0.05 and p < 0.01, respectively, compared between groups as shown. Colors are used for ease of reading. As indicated at the bottom of panels E and F, IL-1α is depicted as black, IL-1β as pink, IL-6 as pale purple, TNF-α as blue, and IL-10 as white. Mean ±95% CI is shown.

Mixed-model analyses showed differences by *group* (p < 0.0001, F_2,55_ = 14.79), *cytokine* examined (p < 0.0001, F_1662,73_ = 27.96), and their interaction (p < 0.0001, F_8,176_ = 8.30) in serum. *Post hoc* analysis revealed that serum IL-1α, IL-6, and TNF−α were elevated in task versus control rats; similarly, serum IL-1α and TNF-α were elevated in trained + rest versus control rats (Figure 4A). However, serum IL-6 was elevated in task versus trained + rest rats (Figure 4A). IL-10 showed no group differences. Of all the cytokines measured, the highest levels were observed for IL-6 and TNF-α (compared to other cytokines: p < 0.01 each).

In forearm flexor digitorum muscles, there were differences by *group* (p = 0.0001, F_2,33_ = 12.69), *cytokine* (p < 0.0001, F_1167,22_ = 130.2), and their interaction (p < 0.0001, F_8,74_ = 13.57). *Post hoc* analysis revealed elevations in muscle IL-1α in task versus control rats, IL-6 in task rats versus both other group, and reduced levels of IL-10 in trained + rest and task rats versus control rats (Figure 4B). IL-6 levels were highest relative to other cytokines (p < 0.01 each).

In forearm bones (radius and ulna), there were differences by *group* (p < 0.0001, F_2,39_ = 12.23), *cytokine* (p < 0.0001, F_4,88_ = 34.11), and their interaction (p = 0.0001, F_8,88_ = 4.45). *Post hoc* analysis revealed elevations in bone IL-1α, IL-1β, and IL-6 in task versus control rats (Figure 4C). Only IL-1α was elevated in trained + rest rats compared to control rats. Moreover, IL-6 was elevated in task versus trained + rest rats (Figure 4C). IL-10 showed no group differences. TNF-α and IL-10 levels were lowest, compared to other cytokines (p < 0.0001 each), and did not differ between groups.

In flexor digitorum tendons, there were differences by *group* (p = 0.03, F_2,33_ = 3.93), *cytokine* (p < 0.0001, F_2256, 45_ = 15.07), and their interaction (p = 0.007, F_8,80_ = 2.88). IL-1α levels were elevated in task versus trained + rest rats, and IL-10 levels were reduced in trained + rest rats versus control rats (Figure 3D).

In median nerves, there were differences by *group* (p < 0.0001, F_2,21_ = 121), *cytokine* (p < 0.0001, F_4,72_ = 147.6), and their interaction (p < 0.0001, F_8,726_ = 43.77). *Post hoc* analysis revealed elevations IL-1α, IL-1β, IL-6, and TNF-α in task versus control rats, and IL-1β and IL-6 in task versus trained + rest rats (Figure 4E). IL-10 showed no group differences.

No differences in cytokine concentrations were found between *group* or *group × interaction* in tail bone and tail tendon tissues (tail tendon and bone tissues were combined; Figure 4F). This suggests that changes in cytokines were predominantly limited to tissues involved in performing the task and serum.

### 3.5 IL-6 expression was increased with task in the cingulate cortex

**FIGURE 5:**
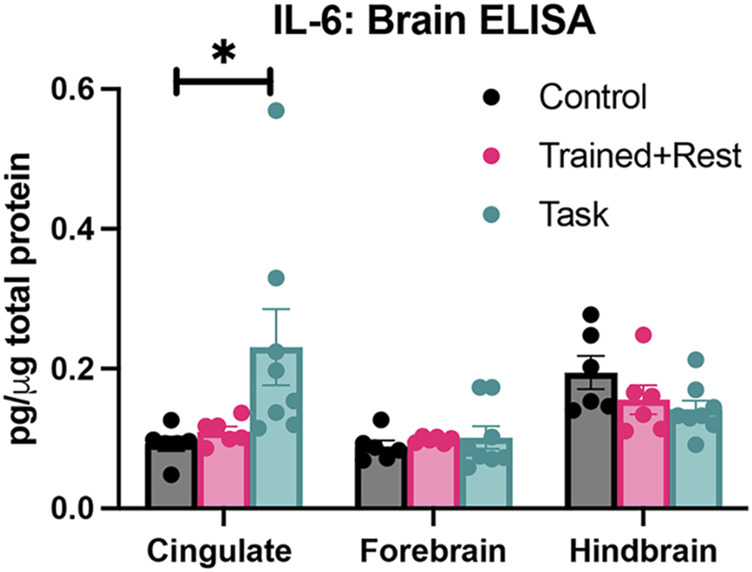
IL-6 in three brain regions (cingulate, forebrain, and hindbrain) collected from each group (control, trained + rest, and task) after euthanasia. Each was tested using ELISA. *p < 0.05, compared between groups.

IL-6 production was also examined in the cingulate cortex, forebrain, and hindbrain regions. Differences were observed by *group* (p = 0.01, F_1409,36_ = 5.34) and region × group (p = 0.005, F_4,51_ = 4.19). Elevated levels of IL-6 was observed in the cingulate cortex of task rats versus control rats (Figure 5). No between-group differences were observed in the forebrain or hindbrain regions.

### 3.6 IL-6 immunostaining was higher in several brain regions in trained + rest and task rats

**FIGURE 6:**
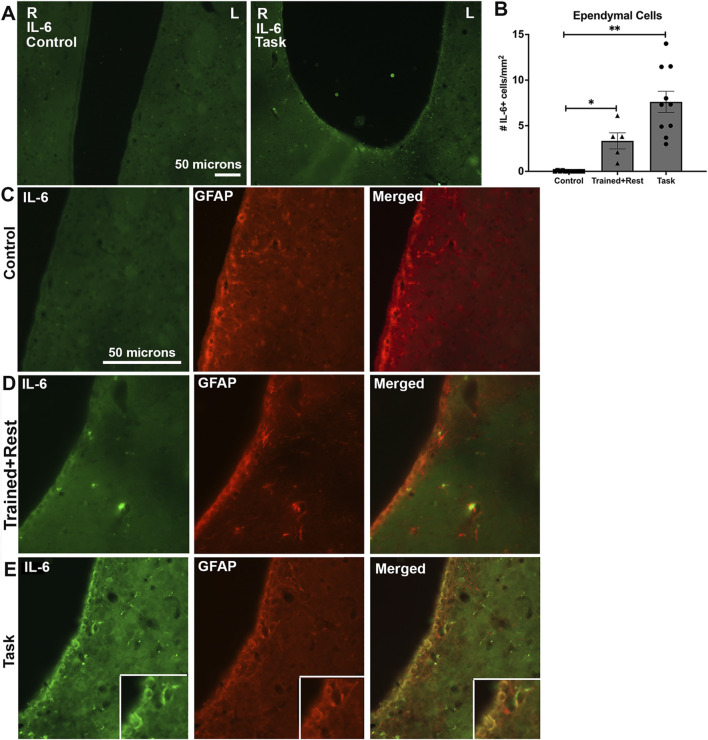
IL-6 (green) and GFAP (red) immunoexpression in ependymal cells lining lateral ventricles. **(A)**Lower-power images of IL-6 immunoexpression in the ependyma linings of the right (R) and left (L) lateral ventricles. Scale bar of 50 microns in the left image showing a control rat image pertains to the right image showing a task rat image. **(B)**Quantification of the number of ependymal cells expressing IL-6. No differences were observed in right versus left sides; therefore, their data were averaged before statistical analysis. **(C–E)**IL-6 immunostaining (green), GFAP immunostaining (red) in the same section, and merged image in control, trained + rest, and task rats, respectively. Insets in E show a 1.3 x enlargement of select cells in larger panels of **(E)**. Mean ±95% CI is shown. *p < 0.05 and **p < 0.01, compared between groups; n.s. = not significant. Scale bar in B’s panels also relates to C’s and D’s panels.

**FIGURE 7:**
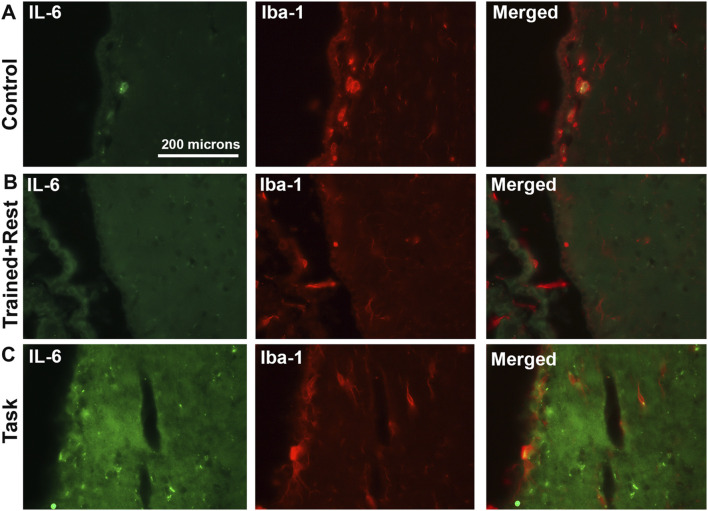
IL-6 (green) and Iba-1 (red) immunoexpression in ependymal cells lining third ventricles. **(A–C)** IL-6 immunostaining (green), Iba-1 immunostaining (red) in the same section, and merged image in control, trained + rest, and task rats, respectively. Scale bar in A’s panels also relates to B’s and C’s panels.

We examined several brain regions for IL-6 immunoexpression on the basis that IL-6 released into the peripheral tissues can act on the brain to induce or alter behavioral responses (Bluthe et al., 2000b; Chang et al., 2024; Xin et al., 2017). IL-6 immunoexpression was higher in ependymal cells lining the lateral and third ventricles in both task and trained + rest rats compared to control rats (Figures 6A–E; Figures 7A–C). No differences were observed in the right versus left sides of the third or lateral ventricles (Figure 6A panels show representative third ventricle images for control and task rats). Few to no IL-6+ cells were observed in the control rat ependyma (Figure 6A, C; Figure 7A). Higher IL-6 immunoexpression was observed in task rat ependyma cells than in control rats, while trained + rest rats showed some increase in the immunoexpression versus controls, although not as much as in task rats (Figures 6D, E). Quantification of the numbers of IL-6+ ependyma cells is shown in Figure 6B and confirms these findings (Kruskal–Wallis p-value, p < 0.0001).

With regard to cell types with immunoexpression of IL-6, in trained + rest and task rats, some IL-6+ cells in the ependyma appeared to be astrocytes, based on their co-localization with GFAP immunostaining (Figure 6E insets). A few IL-6+ cells lining the third ventricle near the paraventricular nuclei of task animals appeared to be microglial cells, based on their co-localization with Iba-1 immunostaining (Figure 7B). This was a rarer site in control and trained + rest rats (Figures 7A, B).

**FIGURE 8:**
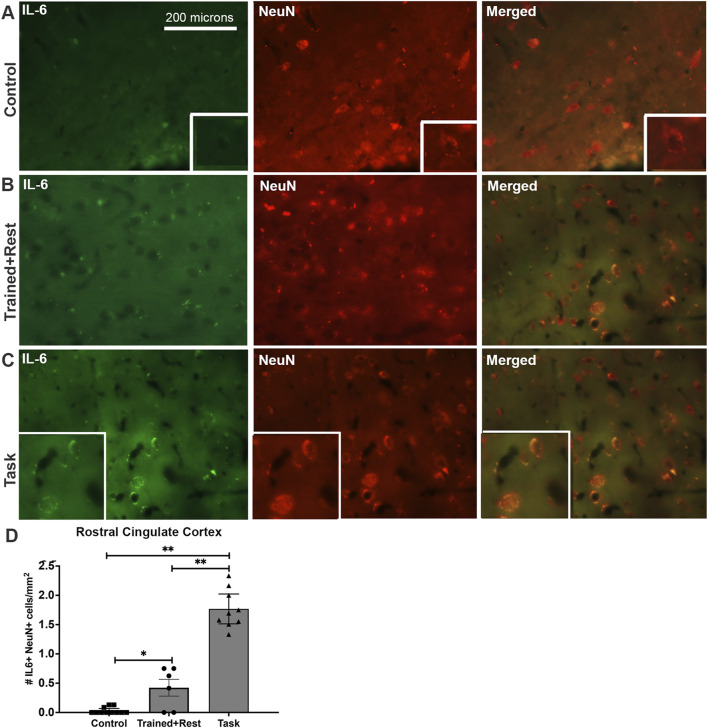
IL-6 (green) and Neu-N (red) immunoexpression in the rostral cingulate cortex. **(A–C)** Control, trained + rest, and task rat brains showing IL-6 (green) and NeuN (red) immunoexpression in the same section and merged image, respectively. **(D)** Quantification of the number of neurons (NeuN+) that co-express IL-6 in the rostral cingulate cortex. Mean ±95% CI is shown. *p < 0.05 and **p < 0.01, compared between groups, as shown. Scale bar in **(A)** also relates to larger images in **(B, C)**. Insets in panels show 1.4 x enlarged images.

The rostral cingulate cortex was investigated due to its potential involvement in pain behaviors and precision grip (Ehrsson et al., 2000). Low yet more IL-6 immunoexpressing neurons (NeuN+) were observed in task rats than in trained + rest and control rats, and in trained + rest versus control rats (Figures 8C, D versus Figures 8A, B, D). This is particularly clear in the insets of enlarged cells shown in the panels of Figure 8A versus Figure 8C. No differences were observed in right versus left sides of the rostral cingulate cortex; therefore, the results were combined for statistical analyses. Quantification of IL-6+/NeuN+ cells in each group confirmed these observations (Figure 8D; Welch’s ANOVA p-value, p < 0.0001).

**FIGURE 9:**
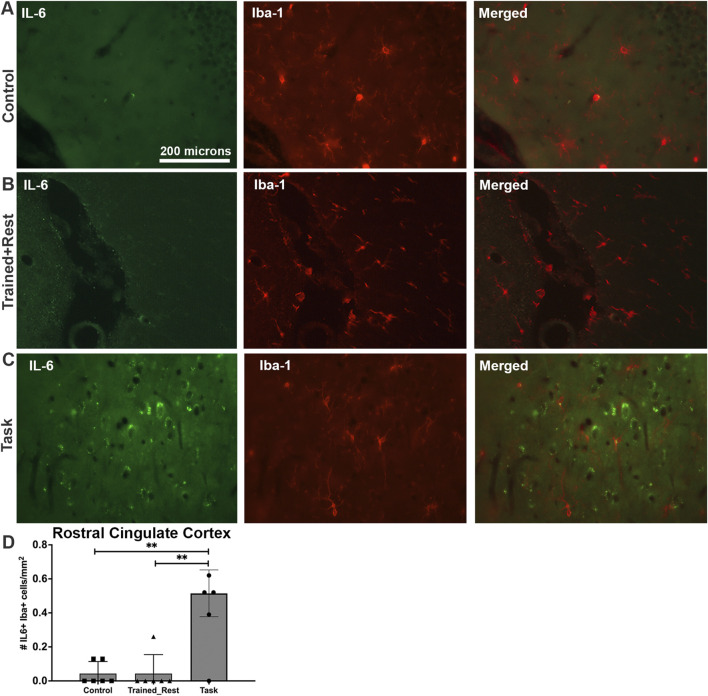
IL-6 (green) and Iba-1 (red) immunoexpression in the rostral cingulate cortex. **(A–C)** Control, trained + rest, and task rat brains showing IL-6 (green) and Iba-1 (red) immunoexpression in the same section and merged image, respectively. **(D)** Quantification of the number of Iba+ glial cells that co-express IL-6 in the rostral cingulate cortex. Mean ±95% CI is shown. **: p < 0.01, compared between groups, as shown. Scale bar in **(A)** also relates to images in **(B, C)**.

A small number of the IL-6+ cells in the rostral cingulate cortex of Task animals were microglial cells, based on their co-localization with Iba-1 immunostaining, although not in the rostral cingulate cortex of either Control or Trained + Rest rats (Figures 9A, B versus Figure 9C). Quantification of IL-6+/Iba-1+ cells in each group confirmed this observation (Figure 9D; Welch’s ANOVA p value, p = 0.03).

**FIGURE 10:**
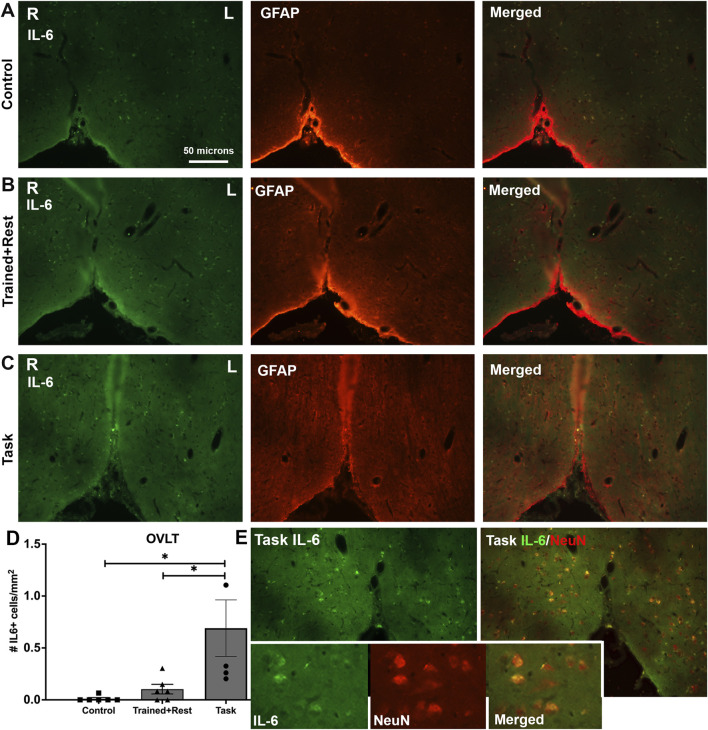
IL-6 immunoexpression in the OVLT. **(A–C)** Control, trained + rest, and task rat brains showing IL-6 (green) and GFAP (red) immunoexpression in the same section and merged image, respectively. **(D)** Quantification of the number of IL-6+ cells in the OVLT. Mean ±95% CI is shown. *p < 0.05, compared between groups. **(E)** IL-6 immunoexpression in task rats (left image) and IL-6+NeuN+ (right image). Insets in panel **(E)** show 1.72 x enlarged image from the larger images of panel **(E)** and depict IL-6 immunoexpressing neurons (NeuN+). Scale bar in **(A)** also relates to larger images in panels in **(B–E)**.

**FIGURE 11:**
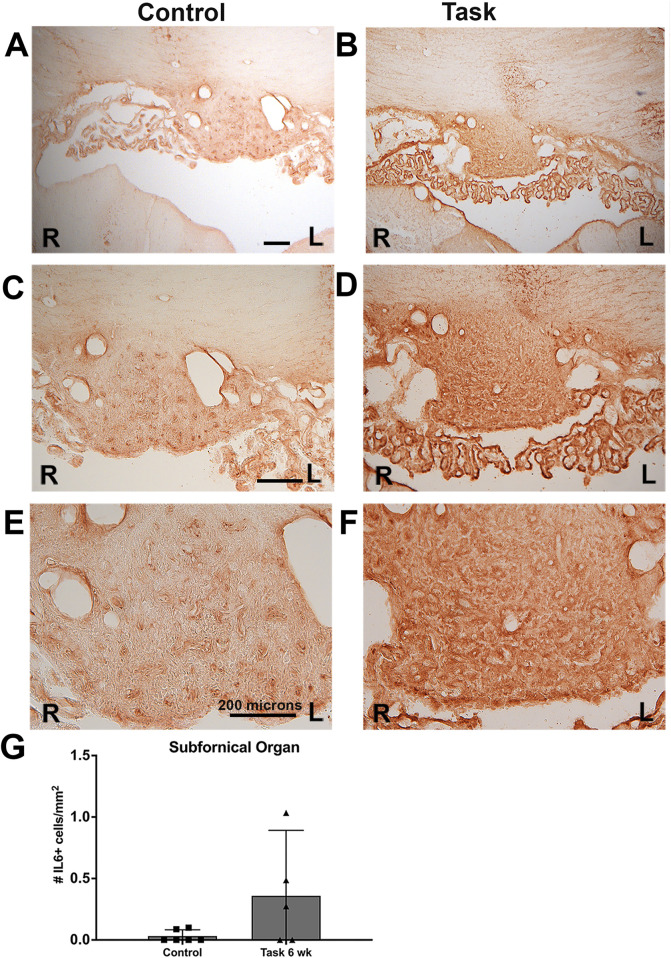
IL-6 immunoexpression in the subfornical organ (SFO) of control and task rats. IL-6 was detected using HRP-DAB (brown). SFO is a mid-line structure; the right (R) and left (L) sides are indicated in each panel. **(A, C, E)** Images from a control rat’s SFO, shown in increasing magnification. **(B, D, F)** Images from a task rat’s SFO, shown in increasing magnification. **(G)** Number of SFO+ cells with IL-6 immunostaining. No differences were observed in right versus left sides; therefore, their data were averaged before statistical analysis. No overall group difference was observed. Scale bar shown are 200 microns; scale bars in the left panels relate to right panels.

Three circumferential organs were examined: the organum vasculosum of the lamina terminalis (OVLT; Figure 10), subfornical organ (SFO; Figure 11), and median eminence. Very few IL-6+ cells were observed in the OVLT of task rats. Yet, this number was higher than that observed in control and trained + rest rats (Figures 10A–C). Quantification of IL-6+ cells in each group confirmed this observation (Figure 10D; ANOVA p = 0.01; no differences were observed in the right versus left sides in the OVLT of any group). Few, if any, of the IL-6 immunoexpressing cells in the OVLT were GFAP+ astrocytes (Figures 10A–C). Therefore, subsets of sections from task animals were co-immunostained with IL-6 and NeuN; the staining showed the presence of IL-6 in several neurons in the OVLT of task rats (Figure 10E and insets).

There was also a greater density of IL-6 immunoexpression in the SFO of Task rats, relative to Control rats (Figures 11A–F), although this did not meet statistical significance (Figure 11G). No right versus left differences were observed in the SFO of any group (Figures 11A–F).

The median eminence showed no training or task responses to IL-6 immunostaining (data not shown).

### 3.7 Correlations between circulating and tissue cytokine levels

As shown in Table 1, circulating (serum) TNF-α levels correlated positively with serum levels of IL-1α and β. As shown in Table 2, serum levels of all cytokines, except for IL-1β, correlated with their levels (i.e., the same cytokine in other tissues) in musculoskeletal and median nerve tissues. Serum IL-1α and IL-6 levels correlated positively with their levels in the flexor digitorum muscle, forearm bones, and median nerve, but not with those in tendons. In contrast, serum TNFα only correlated positively with its levels in the median nerve. IL-1β levels in serum did not correlate with its levels in any tissues.

### 3.8 Correlations between cytokines and behaviors

Table 3 presents the correlations between each cytokine in every tissue examined, except for the brain (i.e., serum, each musculoskeletal tissue, and the median nerve) and each behavior examined. Grip strength is commonly interpreted as a metric of pain-like behavior, especially in response to reaching and pulling tasks, as performed here (Marinus and Van Hilten, 2006; Hadrevi et al., 2019). Declines in grip strength correlated with higher levels of: 1) IL-1α in serum, muscles, and bones; 2) IL-1β in nerves; 3) IL-6 in serum, muscles, bones, and nerves; and 4) TNF-α in the median nerve. Surprisingly, greater grip strength correlated with higher levels of IL-1α and IL-1β in tendons. Forepaw withdrawal threshold is another commonly used metric for pain evaluation in rodents (Tal and Bennett, 1994). Declines in forepaw withdrawal thresholds correlated with elevated levels of each cytokine in the median nerves and IL-6 in serum, but not with any other cytokine in other tissues. With respect to the “sickness” behaviors assayed, less time spent socially interacting with the juvenile rat in a positive manner correlated with higher levels of IL-1α in bone, and IL-6 in serum and muscle. Conversely, greater incidence of aggression correlated with higher levels of IL-1α in bone and nerves, and IL-6 in serum and muscle.

Table 4 presents correlations between brain immunoreactivity and each behavior examined. Both declines in grip strength and forepaw withdrawal thresholds correlated with higher numbers of IL-6+/NeuN+ cells in the rostral cingulate cortex and IL-6+ cells in the ependyma. Less time spent socially interacting in a positive manner correlated with higher numbers of IL-6+/NeuN+ cells in the rostral cingulate cortex and IL-6+ cells in the ependyma. No significant correlations between the incidence of aggression and IL-6 immunoexpression in the brain were identified, although a trend toward a correlation was observed between the incidence of aggression and IL-6 immunoexpression in the cingulate cortex (r = 0.49, p = 0.07).

## 4 Discussion

This study sought to determine if mature rats developed pain-related and sickness behaviors following overuse injury and whether these behaviors correlated with inflammatory cytokine levels in musculoskeletal tissues, serum, and the brain. Following the initial training period, grip strength and forepaw withdrawal thresholds decreased in trained + rest and task rats. For rats that underwent the 6-week rest break after the initial training to high force levels (trained + rest rats), forepaw withdrawal thresholds had returned to baseline levels, although lower grip strength persisted. For rats that performed the high-repetition low-force task for 6 weeks following this training, lower grip strength and forepaw withdrawal thresholds persisted, task performance difficulties and aggressive behavior increased, and the duration of positive social interactions decreased. Several inflammatory cytokines were elevated in response to training and task performance in tissues and serum. Of these cytokines, IL-6 was consistently elevated in task rats in all musculoskeletal and nerve tissues examined, except for forelimb tendon and tail tissues. These elevated serum IL-6 levels were accompanied by elevated IL-6 in brain regions where cytokines can cross the blood–brain barrier (ependyma and OVLT) (Poon et al., 2013; Dantzer, 2001; Dantzer and Kelley, 2007) and a region involved in pain-like and sickness behaviors (the rostral cingulate cortex) (Xiao and Zhang, 2018). Crucially, IL-6 levels in serum and some tissues, including the brain, correlated with all behavioral outcomes. These combined findings suggest that upper-limb WMSDs can drive inflammatory changes locally, systemically, and in brain regions known to contribute to persistent pain and sickness behaviors.

Following training to high-force (55% MPF) pulling, only a few task rats were able to consistently pull at the target 15% MPF across the 6 weeks of task performance (Figure 2). This is consistent with observations from our prior studies showing that training or performing tasks at high force levels induces tissue injuries that contribute to sensorimotor behavioral declines (Barbe et al., 2013; Fisher et al., 2015). We have previously shown that mature rats that are first trained to low-force (rather than the high force used here) pulling before performing a similar high-repetition low-force task for 12 weeks, develop histopathological changes in their tissues, including epi-tendon fibrosis, enthesis disruption at the shoulder, forearm bone loss, and myelin disruption in the median nerve (Elliott et al., 2010; Kietrys et al., 2012; Massicotte et al., 2015; Patel et al., 2024). However, age-related sarcopenia, which typically increases with age (Roubenoff et al., 1998a) and with excessive repetitive motion (Fujiwara et al., 2017), did not contribute to the observed performance declines in this study, based on the cross-sectional widths of flexor digitorum muscles not differing between groups and grip strength remaining strong in age-matched mature control animals across the duration of the experiment.

Injury-induced elevations in tissue and serum cytokines likely contributed to the development of negative spontaneous and evoked behaviors. We and others have reported strong links between inflammatory responses and decreased sensorimotor function both in patients with WMSDs (Gold et al., 2016; Carp et al., 2007; Gold et al., 2014; Gold et al., 2017) and in our rat model of WMSDs in studies that have reported the rescue of many of these changes with administration of anti-inflammatory drugs (Xin et al., 2017; Abdelmagid et al., 2012; Rani et al., 2010; Driban et al., 2011; Jain et al., 2014; Kietrys et al., 2011). It is important to note that the injury model employed herein is operant in nature; thus, increases in local circulating cytokine levels likely originated from the injured tissues (Elliott et al., 2010; Xin et al., 2011; Kietrys et al., 2012; Massicotte et al., 2015) and were not the consequence of experimental injections of inflammatory cytokines, lipopolysaccharides (LPS) or *E. coli*, or other immune-stimulating agents (Barrientos et al., 2006; Campisi et al., 2003; Castanon et al., 2004; Godbout et al., 2005; Huang et al., 2008). That IL-1α and IL-6 levels in serum correlated with their levels in muscle, bone, and nerve, and were generally higher in task rats relative to trained + rest rats, supports this concept. The only tissues that did not express cytokines at greater concentrations were those in the tail, which were not used in lever pulling. Despite the 6-week rest period enforced on trained + rest rats, several pro-inflammatory cytokine levels remained elevated in serum and some tissues. This suggests that the rest period was not long enough to fully resolve all structural or immune changes, which is consistent with prior work showing that repetitive strain-induced histopathologic changes were not completely reversed after 3 months of rest (Stauber, 2004; Stauber and Willems, 2002).

That IL-6 was consistently elevated across tissues/serum in task animals is important because of its key roles in behavior, development, immunity, inflammation, and metabolism. IL-6 is a unique cytokine produced by immune cells, vascular endothelial cells, adipocytes, and skeletal muscle, with both pro- and anti-inflammatory properties (Kishimoto, 2006; Rose-John, 2020). Crucially, excessive and dysregulated IL-6 production is a risk factor for various diseases and disorders (e.g., cardiovascular disease) and often correlates with symptom severity (including pain intensity and declines in physical and cognitive function) (Sharma and Singh, 2014; Singh and Newman, 2011; Carp et al., 2008; Klyne et al., 2017). IL-6 also drives other inflammatory factors (e.g., C-reactive protein) that are pertinent risk factors for diseases and conditions, including cancer and lower-back pain (Klyne et al., 2022; Klyne and Hodges, 2020; Farrell et al., 2023; Zhu et al., 2022; Heikkila et al., 2009; Lagrand et al., 2002). In addition, it is known that peripherally released IL-6 can act on the brain to induce or alter behavioral responses (Bluthe et al., 2000b; Chang et al., 2024; Xin et al., 2017).

As the rats used in this study were very mature (18–24 months of age), we considered the possibility of age-related decrements in immune function (a process referred to as immunosenescence or “inflammaging”) in the context of our results. As mentioned in the introduction, pro-inflammatory cytokines in peripheral tissues sensitize nociceptors locally, which can stimulate the release of agents that modulate nociception both locally and within the central nervous system (brain and spinal cord) (Zhang and An, 2007; Abbadie, 2005; Maier and Watkins, 2003; Watkins et al., 2003), and can reach the brain by crossing the blood–brain barrier at specific sites (e.g., ependymal cells, endothelial cells, and circumventricular organs) via retrograde transport from peripheral nerves to their central nervous system targets, by slow diffusion across the blood–brain barrier or by transport of second messengers through nerves or across the blood–brain barrier that then stimulate cytokine expression in the brain (Poon et al., 2013; Dantzer, 2001; Dantzer and Kelley, 2007; Capuron and Miller, 2011; Hoogl et al., 2015; Quan and Banks, 2007; Varatharaj and Galea, 2017). These various modes of enhanced pro-inflammatory cytokine production in the brain have been shown to initiate and drive a range of physiological and behavioral responses that are collectively known as “sickness behaviors” (Dantzer et al., 1998; Henry et al., 2009; Kelley et al., 2003; Roth et al., 2021; Poon et al., 2013; Parnet et al., 2002), including enhanced pain behaviors, reduced social interaction, and aggression (Zhang and An, 2007; Bluthe et al., 2000a; Bluthe et al., 2000b; DeLeo and Yezierski, 2001; Queiroz et al., 2017; Dantzer et al., 2008; Zalcman and Siegel, 2006; Suarez et al., 2002), as shown in this study. We have also previously shown that IL-1β and TNF-α increase in brain ependymal cells of young adult rats performing a high-intensity task for 6–12 weeks, in parallel with enhanced pain and aggressive behaviors, and reduced social interaction (Xin et al., 2017). Provision of rest or anti-inflammatory doses of ibuprofen as secondary interventions weeks after task onset rescued the aggression and social interaction changes (Xin et al., 2017). However, mechanical allodynia, reach performance, and grip strength were not improved by secondary interventions of ibuprofen, anti-TNF-α, or manual therapy provided after commencement of spinal cord central sensitization changes (increased substance P, neurokinin 1 receptor, TNF-α, and IL-1β) (Xin et al., 2017; Kietrys et al., 2011) or fibrotic forelimb tissues changes (Rani et al., 2010; Barbe et al., 2021). Instead, preventive treatments provided earlier, such as during the initial training period, were necessary to block the development of pain and related responses (Xin et al., 2017; Kietrys et al., 2011; Bove et al., 2019; Bove et al., 2016). Combined, these data support the notion that inflammatory processes are necessary for the development of these behavioral declines, but that central nervous system sensitization is involved in their persistence.

There are conflicting and inconsistent data as to whether indices of inflammation (e.g., cytokine production) increase, decrease, or remain unchanged with aging in humans and animals (Beharka et al., 2001; Krabbe et al., 2004). In human studies, IL-6, but not IL-1β or TNF-α, production by peripheral mononuclear cells is increased in aged subjects, compared to young subjects (Roubenoff et al., 1998b). Alternatively, TNFα and IL-1β production in whole-blood supernatants following *in vitro* LPS stimulation was lower in samples from elderly versus young subjects (Bruunsgaard et al., 1999). However, several studies report no age-related increases in TNF-α or IL-6 (Beharka et al., 2001; Peterson et al., 1994). For example, serum levels of IL-6 were not different in a group of “strictly healthy” individuals over 65 years of age compared to those in a group of 20–30-year-olds (Beharka et al., 2001). We have reported several age-related responses in our rat model. In one study, we reported that mature rats had significantly higher serum levels of IL-1β and IL-6 relative to young adult rats performing the same high-repetition low-force task for 12 weeks, yet no serum inflammatory cytokine differences between control mature versus control young adult rats (Xin et al., 2011). Greater serum IL-1β and IL-6 levels correlated moderately and negatively with declines in grip strength (Xin et al., 2011). In another study examining the effects of a 12-week high-repetition low-force task on nerve and spinal cord tissues, TNF-α increased significantly in the median nerve and cervical spinal cord sensory and motor neurons (as did IL-1β) (Elliott et al., 2010). These increases were concomitant with declines in grip strength and enhanced forelimb and hindlimb mechanical sensitivity in very mature task rats. Again, declines in grip strength were persistent in trained + rest rats (even though the training was to low force only in that study rather than the training to high force used in this current study), while mechanical sensitivity was ameliorated by 12 weeks of rest (Elliott et al., 2010). In a third study examining age-related bone responses to repetitive task performance, IL-6 was elevated in forelimb bones of mature task rats alone, compared to in mature control or young adult task rats, whereas both IL-1β and TNFα levels were more elevated in mature rats than in young adult rats, with or without task performance (Massicotte et al., 2015). In a fourth study examining age-related response differences in tendons, IL-1β and IL-6 levels were higher in flexor forelimb tissues of mature versus young adult task rats in parallel with reduced forelimb motor function (Kietrys et al., 2012). We suspect that the differences in study results (ours and others) could reflect heterogeneity in the age of subjects, species examined, and differential responses between the tissues examined in response to different stimuli (e.g., injury, disease, and disorder).

Numerous animal and human studies have investigated the role of cytokines in sickness responses. For example, previous research links inflammatory cytokines, e.g., IL-1, to reduced motivation and anhedonia (Merali et al., 2003), which could partially explain the reduced task participation over time in this study. Low-dose LPS injections induce small but significant increases in circulating levels of IL-1β and IL-6, which are accompanied with anhedonia and depressive-like behaviors (Teeling et al., 2007). Moreover, patients with viral infections or chronic inflammation have elevated circulating levels of cytokines (e.g., IL-1 and IL-6) that correlate or present with acute sickness behaviors, including fever, malaise, pain, fatigue, mood fluctuations, depressive symptoms, reduced activity, loss of interest in social activities, and even increased aggression (Zalcman and Siegel, 2006; Felger, 2018; Slavich and Irwin, 2014; Dowlati et al., 2010; Vollmer-Conna et al., 2004; Dantzer, 2023). Although systemic inflammatory responses triggered by upper-extremity WMSDs may not be as severe as in some conditions, our data suggest that even lower-grade inflammatory responses that occur with common WMSDs can induce sickness behaviors (Eisenberger et al., 2017; Maes et al., 2012; Kubera et al., 2011).

We also observed elevated IL-6 immunostaining in ependyma cells, rostral cingulate cortex, and both circumferential organs (OVLT and SFO). IL-6 can produce either deleterious or beneficial effects on neuronal function depending on its concentration (Li et al., 2009), and may play a role in the pathogenesis of neurodegenerative disorders (Sawada et al., 2006). Elevated IL-6 level in brains is thought to facilitate reduced food intake, inhibition of memory and learning, neurodegeneration, and numerous other sickness behaviors induced by other cytokines (Godbout and Johnson, 2004). Similarly, we suspect that elevated IL-6 in the brains of task rats is a mechanism underpinning the worsening pain-related and social behaviors. This effect might be enhanced with advanced age. For example, one theory suggests that the physiological balance between pro- and anti-inflammatory cytokines becomes lost with advanced brain aging (Viviani and Boraso, 2011). This translates to the enhanced and persistent production of pro-inflammatory cytokines such as IL-6, IL-1, and TNF (Huang et al., 2008; Sawada et al., 2006; Godbout and Johnson, 2004; Ye and Johnson, 1999). Evidence also points to an increasing proportion of immune cells and glia becoming “reactive” or “primed” with advanced aging (Godbout et al., 2005; Godbout and Johnson, 2004; Godbout and Johnson, 2009), and that once primed, they are more easily triggered and react excessively by overstimulating cytokine production. Further work is needed to establish the role of aging in brain inflammation in response to peripheral tissue injury since this is the first study of this kind.

Grip strength was the only behavioral decline present in both trained + rest and task animals at the end of the study. We suggest that this was the result of elevated IL-6 in both muscle and brain ependyma at the same time-point, although the continued increase in IL-1α and β levels in the median nerve may also have contributed to the grip strength declines since this nerve innervates hand muscles involved in gripping movements. We had anticipated elevated IL-6 within the rostral cingulate cortex since functional magnetic resonance imaging (fMRI)-detected imaging has been shown as high in this cortical area in human subjects engaged in precision gripping (Ehrsson et al., 2000). Perhaps, earlier elevations in the rostral cingulate cortex of trained + rest rats had declined after the 6 weeks of rest, or perhaps the continued elevation of IL-6 in the peripheral tissue is the key contributing factor since grip strength is strongly influenced by cytokine levels (Schafers et al., 2003; Felicio et al., 2014).

This study has several strengths. This is a translational model of WMSDs in which the phased training (6 weeks of 55% maximum pulling strength on a lever bar) followed by a repetitive task (6 weeks at 15% of maximum pulling strength on a lever bar) effectively simulates occupational injury patterns. We performed a multi-system analysis, with simultaneous assessment of IL-6 levels in affected musculoskeletal tissues, serum, and several brain regions to provide a more holistic assessment than typically performed. Multiple behaviors were assessed—grip strength for motor function and pain (muscle pain, i.e., myalgia function), mechanical allodynia for mechanical sensory processing, and psychosocial testing via social interaction tests (affective behavior)—in order to provide a broad outcome matrix. We explored longevity (resolve versus persist) of these behavioral responses over time in response to training and then rest versus task performance by comparing trained + rest versus task rats. Lastly, we included tail tissue analyses since tail tissues are not involved in performing the task.

Two limitations of this study need to be considered. We did not examine rats for febrile changes that might have occurred with increased levels of circulating IL-6, which has been reported in rats following the stimulation of IL-6 in response to LPS injections (Cartmell et al., 2000). However, fever is not a typical characteristic of WMSDs (Barr et al., 2004). Second, only mature female rats were included. As the force transducer sensitivity of our model setup is currently tailored to the pulling strength of female rats, the inclusion of male rats would have reduced data quality and made the interpretation of findings more difficult. We also focused on females because human females have a higher incidence of work-related musculoskeletal disorders than men (Collins and O'Sullivan, 2010; Cote, 2012; Gerr et al., 2002). Future studies are encouraged to include males to contribute to our understanding of sex differences in the development of these disorders. Nevertheless, we have previously determined that there were no significant changes in estradiol levels in rats of this age performing a similar task (Massicotte et al., 2015).

## 5 Conclusion

This study provides first evidence that tissue damage associated with overuse injury can induce inflammatory responses within the local/damaged tissues, circulation, and brain regions that drive pain-related and sickness behaviors. This has potential future implications for the management of not only overuse injuries but also a broad spectrum of musculoskeletal and inflammatory-based conditions. A more complete understanding of the interrelationships between immune responses at different levels (e.g., local tissue vs. systemic vs. spine vs. brain), function/task performance, and sickness behaviors following overuse injury would help direct effective workplace and clinical management strategies.

## Data Availability

The datasets presented in this study can be found in online repositories. The names of the repository/repositories and accession number(s) can be found in the article/Supplementary Material.
