# Supplementary material for: Overuse injury induces persistent behavioral declines that correlate with higher IL-6 expression in the affected musculoskeletal tissues, circulation, and brain
Source: Front Physiol. 2025 Jul 16;16:1500795. doi: 10.3389/fphys.2025.1500795 (PMC12307325; doi:10.3389/fphys.2025.1500795)
Supplement: Supplementary file 1 [file DataSheet1.pdf]

# Supplementary Material

## Repetitive Strain Injury Induces Persistent Grip Strength Declines That Correlate with Increased IL-6 in Serum, Musculoskeletal Tissues, Nerve and Brain

**Mary F Barbe[1*], Alex G. Lambi[2], Parth R Patel[1], Istvan P. Tamas[1], Elizabeth R McGonagle[1], Megan Van Der Bas[1], Betsy A Kalicharan[1], Lewis Holt-Bright[1], Steven N Popoff [1], David M Klyne[3]**

*** Correspondence:** Mary F Barbe, Mary.barbe@temple.edu

## 1 Supplementary Figures

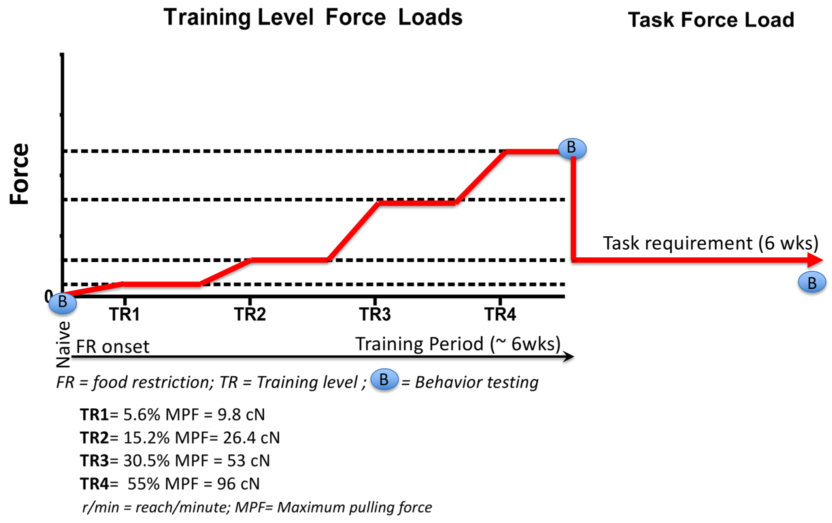


**Supplemental Figure 1.** Diagram showing target force requirements used during training and task performance. The training (TR) requirements increased progressively across the 6 weeks of training from a negligible pulling force requirement of 5.6% of the rats' maximum voluntary pulling force (MPF) to a high force requirement (55% of their MPF). This progressive increase in force was chosen for the induction of tissue injury by the end of training. After the training period, the target force requirement was dropped to 15% of their MPF. This lower force was chosen so that rats could maintain the "work" at the target reach rate and lever pulling requirements for 2 hr/day, 3 days/week, for 6 weeks. We have previously shown that mature rats are able to perform ta15% MPF pulling job, after training to 15% of their MPF, at a reach rate of approximately 4 reaches/min for up to 12 weeks (Massicotte et al. 2015).

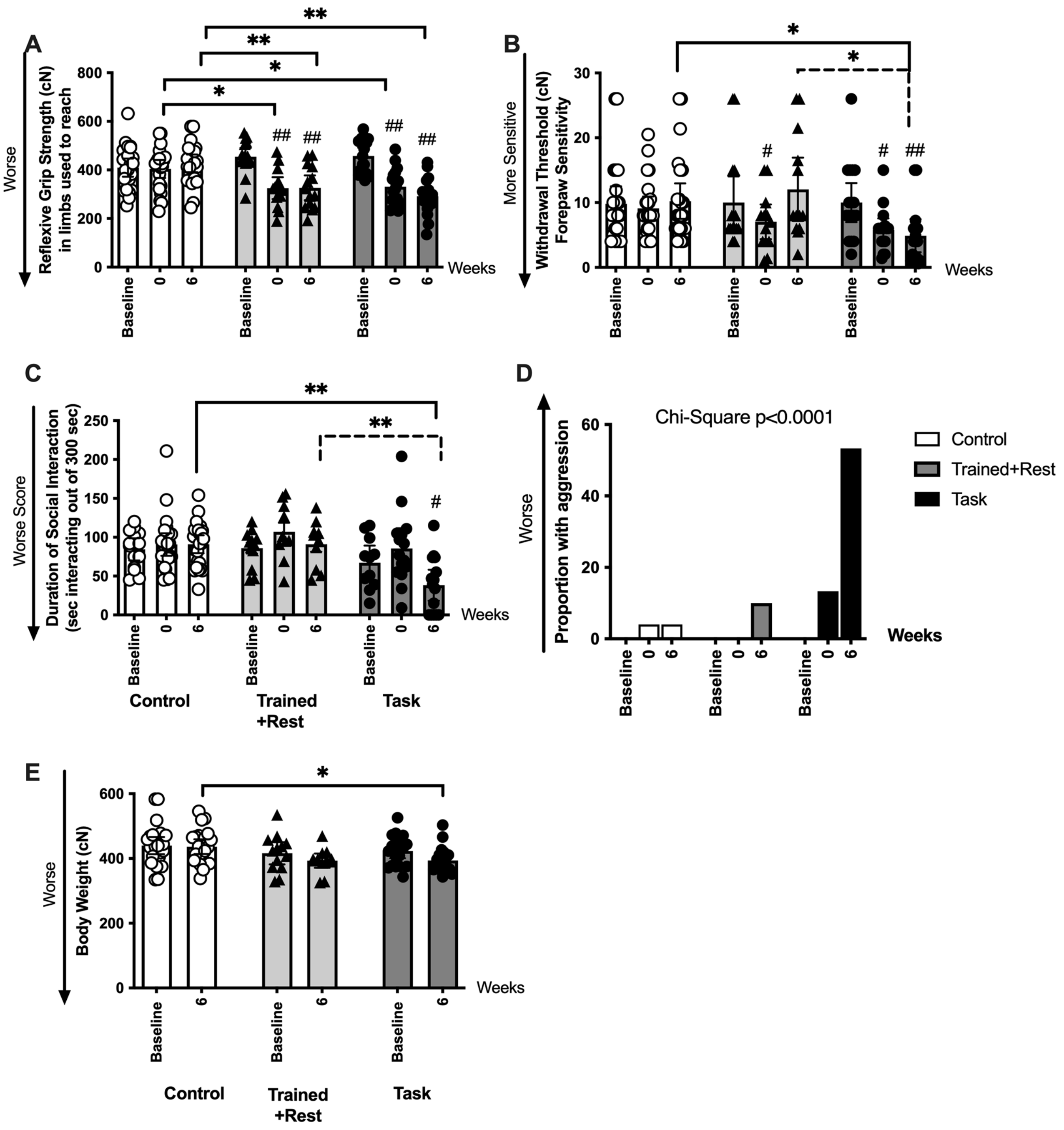


**Supplemental Figure 2.** Pain-related and psychosocial behaviors across time, shown as a scatter dot graph. A) Forearm reflexive grip strength. B) Forelimb mechanical sensitivity, shown as forelimb withdrawal threshold to smallest sized monofilament that elicited a forelimb withdrawal response. C) Duration of social interaction with a novel juvenile rat. C) Proportion of rats showing aggressive behaviors. E) Body weights. Symbols: # and ##: $p<0.05$ and $p<0.01$, respectively, compared to baseline. * and **: $p<0.05$ and $p<0.01$, respectively, compared between groups as shown. Mean ± 95% CI is shown. Mean ± 95% CI is shown.
